# Autoantibodies in the Pathogenesis, Diagnosis, and Prognosis of Juvenile Idiopathic Arthritis

**DOI:** 10.3389/fimmu.2018.03168

**Published:** 2019-01-14

**Authors:** Shawn A. Mahmud, Bryce A. Binstadt

**Affiliations:** Division of Pediatric Rheumatology, Department of Pediatrics, and the Center for Immunology, University of Minnesota, Minneapolis, MN, United States

**Keywords:** autoantibodies, juvenile idiopathic arthritis, antinuclear antibodies, rheumatoid factor, citrullinated self-antigens, anti-citrullinated protein antibodies, carbamylated self-antigens, anti-carbamylated protein antibodies

## Abstract

Autoantibody production occurs in juvenile idiopathic arthritis (JIA) and numerous other autoimmune diseases. In some conditions, the autoantibodies are clearly pathogenic, whereas in others the roles are less defined. Here we review various autoantibodies associated with JIA, with a particular focus on antinuclear antibodies and antibodies recognizing citrullinated self-antigens. We explore potential mechanisms that lead to the development of autoantibodies and the use of autoantibody testing in diagnosis and prognosis. Finally, we compare and contrast JIA-associated autoantibodies with those found in adults with rheumatoid arthritis (RA).

## Introduction

Juvenile idiopathic arthritis (JIA) is a chronic inflammatory disease affecting hundreds of thousands of children worldwide (1). Advances in our understanding of the pathogenesis of JIA over the last two decades have revolutionized therapy, reduced morbidity, and improved quality of life for those affected.

Although often referred to as a single entity, JIA represents a heterogeneous group of inflammatory arthropathies. By definition, JIA is arthritis that begins in a child under the age of 16 years, lasts at least 6 weeks, and is not attributable to any other cause (e.g., Lyme disease, septic arthritis, or “reactive” forms of arthritis). The International League of Associations for Rheumatology (ILAR) has defined seven subtypes of JIA, which are summarized in Table 1 (2). While there are shared genetic and immunologic features between JIA and rheumatoid arthritis (RA) in adults, only a small subset of JIA patients with polyarticular disease and a positive rheumatoid factor (RF) clinically resemble adult RA patients (3, 4).

**Table 1:** Second revision of the ILAR classification of juvenile idiopathic arthritis (2001).

Oligoarticular JIA	Involvement of 1–4 joints in the first 6 months of disease, further defined by the addition of additional involved joints over time (persistent vs. extended)
Polyarticular JIA, RF-negative	Involvement of 5 or more joints in the first 6 months, further defined by absence of rheumatoid factor
Polyarticular JIA, RF-positive	Involvement of 5 or more joints in the first 6 months, further defined by presence of rheumatoid factor
Enthesitis-related JIA	Defined by the presence of arthritis and enthesitis (inflammation of ligamentous and tendinous insertions). Associated with sacroiliitis leading to frequent low-back pain, HLA-B27 positivity, arthritis associated with inflammatory bowel syndrome, and “reactive” forms of arthritis
Psoriatic JIA	Arthritis and psoriasis. Associated with dactylitis, nail changes, and a family history of psoriasis.
Systemic JIA (sJIA)	Arthritis associated with fevers, rash, lymphadenopathy, hepatomegaly, splenomegaly, and/or serositis. Thought to be a systemic auto-inflammatory disease with a distinct pathophysiology as compared to other forms of JIA (10).
Undifferentiated JIA	Chronic idiopathic arthritis which does not fit with one category, or which fits with more than one category above (15–20% of patients)

As we explore in further detail below, although the subtypes of JIA likely differ in their specific pathophysiologic mechanisms, most forms of JIA appear to be rooted in the breakdown of immunologic self-tolerance. Some of the earliest and strongest genetic associations recognized involve the major histocompatibility complex (MHC) class II alleles (4–6), suggesting a critical role for CD4+ T helper (Th) cells. Synovial fluid from inflamed joints in children with oligoarticular, polyarticular, and systemic JIA (sJIA) show an abnormal ratio of Th17 to regulatory T cell subsets, and Th17 cell numbers correlate with arthritis severity (7). Systemic JIA is a distinct subtype driven largely by defects in innate immune mechanisms (8). Interestingly, however, recent work from Ombrello and colleagues highlights the numerous genetic differences between sJIA and the other forms of JIA, yet still identifies the strongest genetic linkage of sJIA as the MHC class II allele DRB1^*^11 (9, 10). These findings support the notion that autoreactive CD4+ T cells are key contributors to the pathogenesis of each of the JIA subtypes.

One way CD4+ T cells contribute to JIA pathogenesis is by providing help to autoreactive B cells. In general, linked T- and B- cell recognition of self-antigens allows CD4+ T cells to promote affinity maturation of B cell clones. Analysis of JIA synovial fluid reveals changes consistent with B cell activation, including alterations in the immunoglobulin light chain repertoire suggestive of secondary V(D)J-recombination, and increased numbers of class-switched memory B cells and plasmablasts secreting IgG within the synovial fluid of affected joints (11–13).

Although autoreactive B cells also have other important pathogenic functions in JIA, such as functioning as antigen presenting cells within the synovium (14), this review focuses on autoantibody production as a consequence of autoreactive B cell activation. Various autoantibodies have been associated with JIA, including anti-nuclear antibodies (ANA), rheumatoid factor (RF), anti-citrullinated protein antibodies (ACPA), and others. In the following sections, we explore the role of autoantibodies in the pathogenesis, diagnosis, prognosis, and response to therapy in JIA.

## Antinuclear Antibodies (ANA): Overview and Use in Diagnosis

The discovery of substances in sera of patients with autoimmunity that can bind to nuclear elements of healthy cells dates back over six decades (15–17). Ultimately these serum factors were shown to be IgG antibodies recognizing nuclear antigens and named antinuclear antibodies (ANA). Modern clinical laboratories detect ANA via an immunofluorescence based assay (FANA, or fluorescent antinuclear antibody test) or an enzyme-linked immunosorbent assay (ELISA). It is now known that several key autoantigens are identified by ANA, including nucleic acids, nucleosomes, phospholipids, and several nuclear and nucleolar proteins (18, 19). These autoantigens are theorized to normally be “hidden” but are exposed to antigen presenting cells during cell death, particularly during apoptosis—a process which has been shown to be abnormal in patients with SLE (20).

The ANA is a highly sensitive test (>95%) for SLE in both adults and children, but it is also commonly misunderstood by clinicians as a general test for autoimmune or rheumatic disease (21–24). Other diseases strongly associated with a positive ANA include mixed connective tissue disorder, juvenile dermatomyositis, Sjogren's syndrome, scleroderma, autoimmune hepatitis, primary biliary cirrhosis, ulcerative colitis, and autoimmune thyroiditis (25–31).

The ANA test is not used to diagnose JIA. However, it has important prognostic value with respect to the risk of uveitis (explored further below). The overall seroprevalence of a positive ANA among all subtypes of JIA combined is < 50% (32). Simply stated, although a positive ANA is more common in children with JIA than among healthy children, the presence or absence of ANA does not change the likelihood that a given patient will have or will develop JIA. Furthermore, false positivity and transient positivity of the ANA (e.g., secondary to infections) are common occurrences (33).

ANA positivity amongst the JIA subtypes is highest in patients with oligoarticular JIA (up to 70%) and is particularly more prevalent in young, female patients (34). Similarly, among patients with psoriatic JIA, ANA positivity is associated with early-onset disease and female predominance (35). ANA positivity is less common in patients with undifferentiated JIA and systemic JIA (32), although a recent study showed patients with systemic JIA have rising ANA and rheumatoid factor titers over time (36).

## ANA and JIA Prognosis

There is mixed evidence in the literature about a potential association between ANA status and arthritis prognosis in patients with JIA. In perhaps the largest study to date addressing this question, there were no significant differences found relating to ANA positivity in Danish children with JIA and the number of active joints at follow-up, remission rate on medication for more than 6 months, or remission rate off medication for more than 12 months. However, patients with RF, HLA-B27, and/or uveitis (either past or present) tended to have lower rates of remission at follow-up, irrespective of ANA status (32). A separate study found that ANA status did not predict risk of relapse when withdrawing tumor necrosis factor inhibitors from patients in disease remission (37).

Another group used a microarray approach to test sera of patients with oligoarticular JIA for reactivity to over 100 autoantigens. A heat map analysis of these arrays was used to perform a cluster analysis and identified two primary groups of patients. Children in cluster 1 were more likely to have high titers of autoantibodies recognizing nuclear antigens such as histone and chromatin, while those in cluster 2 were more likely to have low levels of these autoantibodies, similar to healthy controls. ANA status was not significantly different between these clusters of patients; however, children in cluster 1 were more likely to have active arthritis at follow-up at 5 months (90 vs. 36%; *p* < 0.024) despite there being no significant differences in their treatment regimens (38). This study suggests that autoantibodies directed against more specific autoantigens may be better biomarkers than the ANA with respect to JIA prognosis.

Although the standard ANA test is not particularly helpful in predicting arthritis outcomes in JIA, its prognostic utility for the risk of developing JIA-associated uveitis is clear. Specifically, patients with oligo- or poly-articular JIA with ANA positivity tend to develop disease at a younger age, have asymmetric patterns of arthritis, and are at an increased risk of developing chronic anterior uveitis (39, 40). This form of uveitis is typically asymptomatic. If undetected and untreated, it can result in permanent vision loss. It is therefore recommended that all patients with certain subtypes of JIA and a positive ANA have more frequent screening eye exams (41). Of note, while a positive ANA indicates increased risk of uveitis, it does not seem to be helpful in predicting the timing or severity of this comorbidity (26).

## Rheumatoid Factor (RF): Overview and Role in Diagnosis

Nearly 80 years ago, Eric Waller discovered a serum factor capable of agglutinating of sheep red blood cells, which others subsequently found to be more common among patients with RA (42, 43). This factor is now known as rheumatoid factor (RF) and refers to a group of antibodies of various classes whose antigen binding sites are specific to the Fc portion of IgG molecules. Not surprisingly, RF has a notable capacity to induce false positivity in laboratory assays such as those designed to detect antibody responses to vaccines or infectious pathogens or autoantibodies such as those seen in antiphospholipid syndrome (44).

RF is most commonly associated with RA in adults and is one of the two serologic tests (along with ACPA, discussed below) included in the current classification criteria for RA (45). A recent meta-analysis of adult RA showed pooled values of sensitivity and specificity of 69% (CI, 65–73%) and 85% (CI, 82–88%), respectively (46). RF can also be positive in other autoimmune disorders such as acute rheumatic fever, SLE, and Sjogren's syndrome. It can be seen non-specifically positive in infections such as tuberculosis and Lyme disease, as well as in otherwise healthy individuals (44, 47, 48).

RF was discovered in some patients with juvenile arthritis long ago (49). Although the overall seroprevalance of RF in patients with JIA is very low (<5%), it confers a worse prognosis (50). In particular, patients with RF+ polyarticular JIA are at higher risk of a more aggressive disease course and bone erosion than JIA patients without RF (51–54). RF+ polyarticular JIA has long been recognized to represent the true pediatric version of RA, and genetic analyses confirm this (4, 55). Consensus treatment plans developed by the Childhood Arthritis and Rheumatology Research Association (CARRA) recognize RF (and ACPA, discussed below) as poor prognostic risk factors among patients with polyarticular JIA, leading most pediatric rheumatologists to use more aggressive, early therapy (e.g., TNF inhibitors) for these patients than for patients without RF (56).

Interestingly, RFs play physiologic roles in the normal immune system. RF of the IgM isotype (IgM-RF), for example, promotes phagocytosis and the removal of antigen-antibody complexes in the course of infection, fixation of complement, and enhancing B cell antigen uptake and presentation to CD4^+^ T cells (57). However, these naturally-occurring IgM-RFs are of low affinity and polyreactive, whereas pathogenic IgM-RFs tend to have undergone affinity maturation (58). Although IgM-, IgG-, and IgA-RFs are often elevated in RA, with IgM-RF being the most common, the isotype-switched IgG and IgA classes are felt to be more causally linked to immunopathology and bone erosion (47, 59). The mechanisms that go awry resulting in the production of RF are not entirely understood, but appear to depend on immune-complex recognition by B cell receptors in the context of toll-like receptor stimulation, as well as T cell help (60, 61). Additional roles for RF in arthritis pathogenesis are explored further below.

## Anti-Citrullinated Protein Antibodies (ACPA): Evidence of a Breach in Self-Tolerance

In 1964, work by Nienhuis and Mandema led to the discovery of another class of autoantibodies that recognize post-translationally modified autoantigens, now called anti-citrullinated protein antibodies or ACPA (tested clinically as anti-cyclic citrullinated peptide or anti-CCP). Nienhuis and Mandema studied sera from patients with SLE, RA, and ankylosing spondylitis and found a factor more commonly present in RA patients that stained the cytoplasm surrounding the nucleus “like the rings of Saturn.” They called this “the antiperinuclear factor” (62). Subsequent work showed that anti-keratin antibodies stained with a similar pattern as the antiperinuclear factors derived from patients with RA (63, 64). We now know that a variety of self-proteins including collagen, fibrinogen, vimentin, filaggrin, alpha-enolase, and others are bound by these antibodies (51, 65–73).

Citrullination is a form of post-translational modification in which arginine residues are changed to citrulline. This modification is mediated by a class of enzymes called peptidylarginine deiminases, or PADs. Several isoforms of PADs exist, and PAD2 and PAD4 have been specifically implicated in inflammatory states. Expression of these enzymes, primarily derived from neutrophils, is increased in the synovial fluid of both mice and humans with inflammatory arthritis (74, 75).

PAD enzymes are required for the formation of neutrophil extracellular traps (NETs), a phenomenon in which neutrophils project DNA and histones into the extracellular environment to aid in phagocytosis. PAD4-activity is required for hypercitrullination of histones, which causes heterochromatin decondensation and the unfolding of chromatin, forming NETs (76). This natural innate immune mechanism sets the stage for modification of self-antigens in the context of inflammatory states and can lead to breakdown of immune self-tolerance (77).

**Figure 1:**
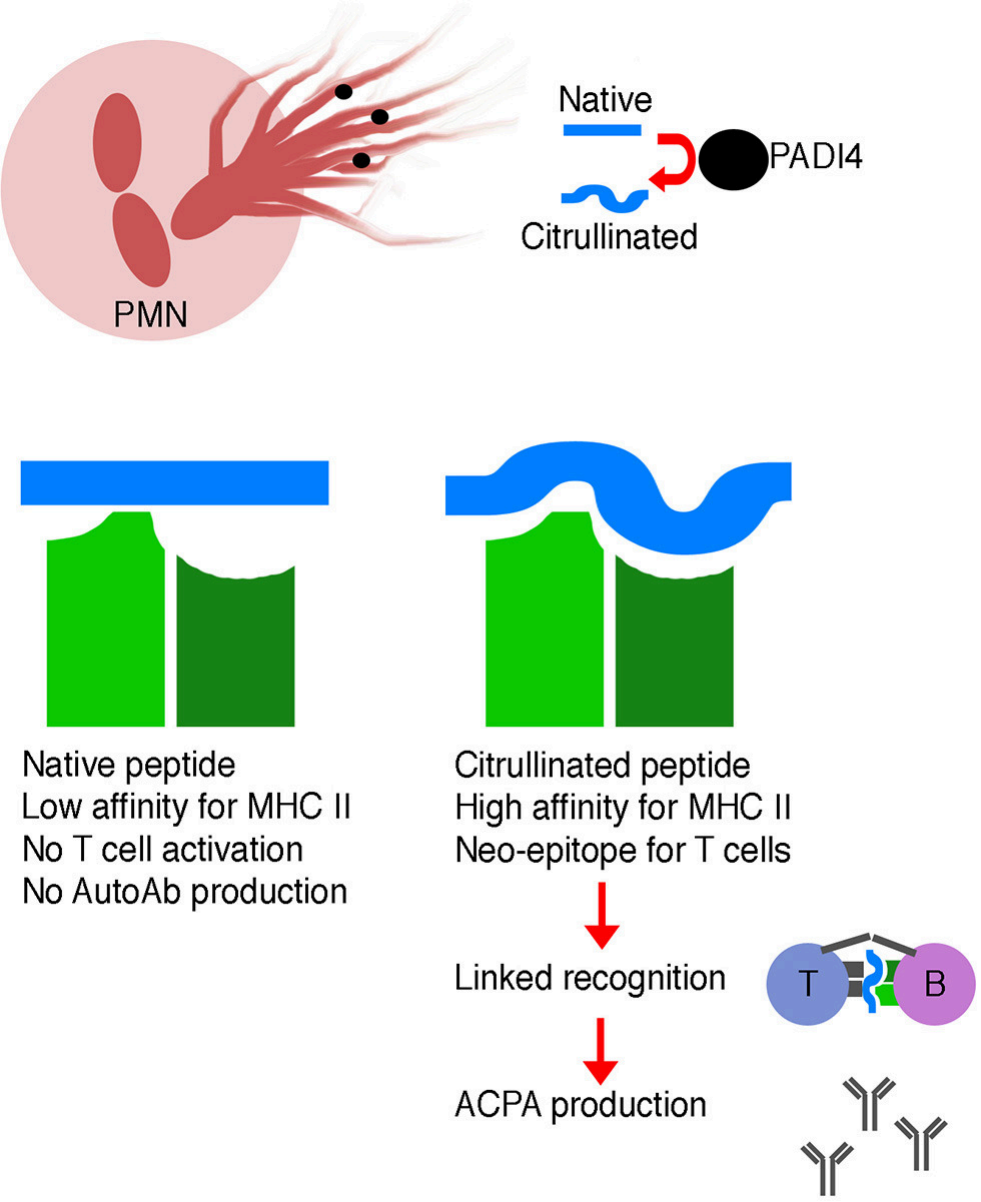
Schematic illustrating the generation of ACPA. Neutrophils are a source of PADI4 enzyme, which leads to citrullination of self-peptides. Citrullinated self-peptides may have altered MHC class II binding affinity, which may result in more efficient presentation to T cells. These modified peptides are neo-epitopes, and T cells specific for these modified peptides may not have been deleted during negative selection in the thymus. Linked recognition of a citrullinated self-antigen and a corresponding citrullinated self-peptide by B and T cells, respectively, stimulates affinity maturation and production of high affinity ACPA.

Normally, thymocytes bearing T cell receptors with high affinity for self-antigens are either clonally deleted or are diverted into the regulatory T cell lineage, reducing the likelihood of autoimmunity—also known as central tolerance (78). However, citrullination of peptides generates neo-epitopes—peptides not present during thymic selection and therefore incapable of inducing central tolerance. Thymocytes specific for these citrullinated neo-epitopes can therefore escape central tolerance and go on to help drive autoantibody production (Figure 1).

Naïve T cells specific for citrullinated autoantigens have the potential to become activated, leading to pathogenic helper T cell responses including those that drive autoantibody formation. Indeed, sophisticated approaches using class II MHC tetramers loaded with citrullinated self-peptides have shown that self-reactive T cells recognizing citrullinated self-antigens exist more commonly among patients with adult RA. Relative to healthy control subjects, RA subjects have similar frequencies of influenza-specific CD4+ T cells, but have ~5-fold increased frequencies of T cells specific for a panel of citrullinated self-peptides (derived from vimentin, fibrinogen, enolase, and cartilage intermediate-layer protein). These cells are predominately Th1 effector-memory cells expressing CD45RO and CXCR3. Treatment of RA patients with biologic therapies (TNF inhibitors, abatacept, or rituximab) restores the numbers of these cells to levels seen in healthy controls, whereas therapy with conventional disease modifying anti-rheumatic drugs such as methotrexate, hydroxychloroquine, or leflunamide) does not (79).

To our knowledge, additional approaches to characterize other effector and regulatory populations of citrullinated self-antigen-specific T cells have not yet been undertaken in either RA or JIA. For example, although the total numbers of follicular T helper cells (Tfh cells) are increased in the peripheral blood and synovial fluid of RA patients (80), their antigenic specificity is not known. Tfh cells drive germinal center B cell affinity maturation and are hypothesized to play a key role in autoantibody generation. One would predict that since the numbers of Tfh cells correlates with ACPA titer in RA patients (81), at least a fraction of these cells should be specific for citrullinated self-antigens. Moreover, a population of regulatory T cells co-expressing both FOXP3 and IL-17 has been observed to accumulate at the sites of inflamed joints in RA patients (82). Future studies should focus on defining the antigen specificity of these Tfh and Treg cells, focusing on citrullinated self-antigens.

## ACPA: Use in Diagnosis and Prognosis of JIA

Children with RF+ polyarticular JIA also commonly have positive ACPA tests (83, 84). This is particularly true amongst patients with the HLA-DRB1^*^0401 (DR4) and HLA-DRB1^*^0101 (DR1) haplotypes. As earlier studies have pointed to marked similarity between these patients and adult RA patients, it is not surprising that there is a high frequency of ACPA in adult RA patients that express the same MHC class II molecules (85–87). ACPA are highly specific for adult RA, and have been shown to predict future risk for developing RA in otherwise healthy individuals (88). As with RF, the sensitivity of ACPA for detecting JIA is very low, but in patients with RF+ polyarticular JIA, these autoantibodies are highly specific (68, 84, 89–95).

The presence of ACPA in polyarticular RF+ JIA has been shown by numerous studies to confer a greater risk of more aggressive and erosive disease (51, 84, 85, 90, 93, 95–99). This is mirrored in patients with ACPA-positive adult RA, who also have a more severe disease course (98, 100, 101). As with RF, ACPA positivity among children with polyarticular JIA typically leads pediatric rheumatologists to recommend earlier, more aggressive therapy (56).

## RF, ACPA, and Circulating Immune Complexes: Potential Roles in the Pathogenesis of Inflammatory Arthritis

Whether or not ACPA have a direct role in the pathogenesis of inflammatory arthritis is unclear. ACPA- and fibrinogen-containing immune complexes have been shown *in vitro* to induce macrophage TNF production via binding to Fc-gamma receptor IIa (102, 103). ACPA have also been shown *in vitro* to activate complement and elicit macrophage activation by crosslinking TLR4 and Fc gamma receptors, and *in vivo*, to enhance tissue injury in murine models of inflammatory arthritis. Importantly, RF and ACPA may synergize to augment some of these functions (104–107).

Circulating immune complexes are elevated in RA and JIA. Immune complexes containing RF, complement factors C1q, C4, C3, C4, and components of the membrane attack complex (MAC) are detectable in synovial fluid of patients with polyarticular RF+ JIA (108). Binding of MAC to circulating immune complexes correlates with erythrocyte sedimentation rate, suggesting that in JIA, complement-mediated tissue damage is induced by the classical complement activation pathway (109).

Given the strong association between RF and ACPA positivity in RA patients, questions about a shared role in pathogenesis are being investigated. A large study of US veterans with RA demonstrated that patients with doubly positive ACPA and RF have higher clinical disease activity scores, serum CRP, and levels of TNF-alpha, IL-1 beta, IL-6, IL-12, and IL-17A. The same authors also performed additional *in vitro* experiments using peripheral blood mononuclear cells from healthy controls incubated with IgG-ACPA containing immune complexes derived from RA patients, and found that the addition of IgM-RF significantly increased TNF-alpha production in monocytes (110). Recently, immune complexes containing both IgG-ACPA and IgM-RF have also been identified in RA patients (111).

Interestingly, anti-PAD4 autoantibodies that increase PAD4 catalytic activity have been discovered in a subset of adult RA patients with particularly erosive disease (112). This indicates a possible feed-forward loop in driving additional self-reactivity against citrullinated autoantigens, and identifies a possible pathogenic role for anti-PAD4 autoantibodies.

Unfortunately, because there are far fewer children with JIA than adults with RA, much of our knowledge about the role of RF, ACPA, and other autoantibodies in inflammatory arthritis is derived from the adult literature. This is clearly a limitation, since these studies only pertain to a small subset of JIA patients overall, specifically children with RF+ polyarticular JIA.

## Autoantibodies Recognizing Other Modified Antigens

Another class of autoantibodies that have recently garnered attention are those that recognize carbamylated self-proteins. Like citrullination, carbamylation is another post-translational modification; in the case of carbamylation, lysine and taurine residues are replaced by isocyanic acid. The phagocyte peroxidase enzymes, myeloperoxidase, and eosinophil peroxidase oxidize thiocyanate, forming two compounds that can result in protein carbamylation: cyanate and hypothiocyanous acid (or HOSCN) (113, 114). Like PAD4, myeloperoxidase is also found in NETs and thus may be involved in promoting carbamylation of self-antigens at sites of neutrophilic inflammation (115).

Interestingly, smoking may be linked to carbamylation. Cigarette smoke contains cyanide, which can be oxidized by myeloperoxidase into cyanate. Cyanate can subsequently participate in non-enzymatic reactions that result in carbamylation under physiologic conditions (116). Exposing mice to cigarette smoke resulted in the generation of carbamylated vimentin, and the sera from exposed mice were broadly reactive against a variety of carbamylated antigens, unlike control mice (117). Smoking and non-smoking RA patients were also studied, and while anti-carbamylated protein antibodies were detectable in both, titers were significantly higher in smokers than in non-smokers (117).

A variety of autoantibodies have now been identified that recognize carbamylated proteins in patients with RA, SLE, and JIA (118–121). A recent study of pediatric rheumatology patients revealed detectable anti-carbamylated protein antibodies in 31% of patients with oligoarticular JIA, 21% of patients with polyarticular-RF positive JIA, in 13% of patients with polyarticular-RF negative JIA, and in 0% of healthy controls (119). Notably, a recent meta-analysis showed that triple positivity for RF, ACPA, and anti-carbamylated protein antibodies was highly specific to patients with active RA, and patients who would go on to develop RA (122).

Additional forms of post-translational modification are relevant to the JIA “peptidome.” For instance, carbonylation is the modification of arginine into glutamic semialdehyde, and proline or methionine residues can undergo oxidation (123). Oxidation has been studied in the context of the molecular chaperone, transthyretin (TTR), which normally functions as a transporter of thyroxine, retinol, and other substances. Both TTR and anti-TTR antibodies are detected in the synovial fluid and plasma in greater concentrations in JIA patients than in controls. TTR can form aggregates when oxidized, and these aggregates were detected in JIA synovium. Interestingly, experiments in HLA-DR1 transgenic mice revealed that *in vivo* T cell proliferation was significantly higher when mice were immunized with oxidized, rather than native TTR, suggesting that the aggregated forms of this autoantigen were more immunogenic (123). Finally, malondialdehyde-acetaldehyde (MAA) adducts are a consequence of oxidative stress; MAA adduction of proteins is increased among patients with RA, and the presence of anti-MAA antibodies correlates with ACPA positivity (124).

While knowledge regarding T and B cell responses to post-translationally modified autoantigens is burgeoning, it is becoming clear that these alterations represent distinct portals for breakdowns in immune self-tolerance, as these modified proteins can serve as neoantigens.

## Autoantibodies and Potential Examples of Molecular Mimicry in RA and JIA

A significant body of research shows an association between periodontal disease and RA, and detectable titers of antibodies to the pathobiont, *Porphyromonas gingivalis*, are seen more commonly in RA patients than controls (125). Alpha-enolase is an established autoantigen in RA which undergoes citrullination and is present in the inflamed synovium (126). Immunization of human HLA-DR4-expressing transgenic mice with enolase derived from *P. gingivalis* induced arthritis and the development of anti-citrullinated enolase autoantibodies. Thus, molecular mimicry between exogenous enolase derived from *P. gingivalis* may be a potential mechanism driving autoantibody formation in RA, particularly in patients with periodontal disease and colonization with *P. gingivalis* (127).

Another example of molecular mimcry which could contribute to the pathogenesis of JIA involves binding immunoglobulin protein (BiP), a member of the heat shock protein (HSP) 70 family, and another established autoantigen in inflammatory arthritis. Approximately 60% of adult patients with RA and 37% of pediatric patients with polyarticular-RF+ JIA have anti-BiP antibodies (128, 129). BiP appears to have immunomodulatory effects, including inducing the production of IL-10, IL-1 receptor antagonist, soluble TNFRII, and downregulation of CD86 and HLA-DR (130). DBA/1J mice immunized with citrullinated-, but not native BiP, developed broadly reactive ACPAs. In a collagen-induced arthritis model, pre-immunizing mice with citrullinated BiP exacerbated the phenotype. The same authors also detected autoantibodies to both native and citrullinated BiP in adult RA patients (131).

Interestingly, a putative mimetope for BiP_336−355_ has been discovered in bacterial HSPs. Immunization of HLA-DR4 transgenic mice with HSP70_287−306_ derived from *Mycobacterium leporae* induced the development of BiP autoantibodies. In the collagen-induced arthritis model, oral administration of this mycobacterial peptide induced tolerance, blunting the development of arthritis and reducing anti-BiP autoantibody production (132). Thus, tolerance against BiP, a common autoantigen in RA and JIA, may be broken by immune responses directed against a mycobacterial pathogen.

## New Approaches to Autoantibody Identification

Admittedly, much of the knowledge of autoantibodies in JIA derives from experience and knowledge in adult RA. While this is not unreasonable, it has major shortcomings since the vast majority of patients with JIA do not fall into the RF+ polyarticular subtype, and are therefore clinically and likely pathophysiologically distinct.

In recognizing this shortcoming, one group applied a novel high-throughput nucleic acid programmable protein array in a small sample of JIA patients to screen for autoantibodies reactive against 768 proteins and identified 18 antibody specificities that could segregate two clusters of patients (133). To our knowledge, the autoantibodies discovered in this study have not been tested in control patients, nor has this approach been applied to a larger sample of patients. However, this or similar methods have the potential to detect additional autoantibody specificities that may promote new insight into the pathogenesis of JIA.

Another group recently looking to develop a novel diagnostic tool for JIA looked to overcome the diversity of autoantibodies in JIA and screened a random library of peptides displayed via Phage-ELISA for mimetopes with reactivity to large percentages of patients with JIA. This method successfully identified a mimetope named “PRF+1” which discriminated JIA patients from controls with a sensitivity of 61% and specificity of 91%. This peptide was subsequently applied to a differential pulse voltammetry system to generate an electro-biochemical sensor for rapid detection of anti-PRF+1 antibodies (134, 135).

## Summary

Autoantibody testing is commonly performed among children with suspected JIA. It is essential to recognize that JIA is much more heterogeneous than RA in adults, and that information regarding the value of particular autoantibodies in adult RA (e.g., RF and ACPA) apply to only a small subset of JIA patients, i.e., those with RF+ polyarticular JIA. We have provided an overview of commonly used autoantibody tests in JIA, seeking to explain their clinical utility as well as limitations and challenges to the field.

The ANA test is a non-specific test of autoantibody reactivity against nuclear antigens. Although highly sensitive for SLE and other related conditions, it is not a diagnostic test for JIA. ANA positivity is most commonly seen in young, female patients with oligoarticular disease. It does not clearly associate with differences in prognosis in any subtype of JIA. Positive ANA status does, however, increase the risk of uveitis and thus its use in clinical practice is primarily focused on predicting the ophthalmologic complications of JIA.

Patients with polyarticular JIA with RF and ACPA positivity have more aggressive and erosive disease. These markers are also not diagnostic tests for JIA, since most JIA patients do not have the seropositive polyarticular subset of disease. Although there are no studies proving roles for RF and ACPA in arthritis pathogenesis, *in vitro* studies indicate that these two groups of antibodies may interact to drive immunopathology.

The multitude of targets recognized by all major classes of autoantibodies in JIA has made determining roles in pathogenesis more difficult. It is likely these roles will not become clear until advances in proteomics allow us to screen more patients for more specific autoantibodies, and by embracing the complexities of these autoimmune responses rather than attempting to simplify them.

## Author Contributions

SM wrote and edited the manuscript. BB helped conceive of and edit the manuscript.

### Conflict of Interest Statement

The authors declare that the research was conducted in the absence of any commercial or financial relationships that could be construed as a potential conflict of interest.

## Abbreviations

JIA: juvenile idiopathic arthritis
ANA: antinuclear antibody
RF: rheumatoid factor
ACPA: anti-citrullinated protein antibody
SLE: systemic lupus erythematosus
RA: rheumatoid arthritis
MHC: major histocompatibility complex
NETs: neutrophil extracellular traps.
